# Regression to Middle Effect May Threaten Validity of Triage Scales; a Letter to Editor

**Published:** 2018-11-18

**Authors:** Amir Mirhaghi, Mohsen Ebrahimi

**Affiliations:** 1 Nursing and Midwifery Care Research Center, Mashhad University of Medical Sciences, Mashhad, Iran; 2 Department of Emergency Medicine, Faculty of Medicine, Mashhad University of Medical Sciences, Mashhad, Iran.

Dear Editor; 

Triage is sorting patients based on acuity in order to manage care in the emergency department (ED) (1). Medical institutes are trying to develop triage scales compatible with their own culture of care. A triage scale must be precise and comprehensive enough to guide triage nurses and eliminate uncertainty. 

It is vital to address the potential pitfalls of the new triage scales to enhance their reliability and validity. Performing a validation study in other institutes provides a great opportunity for medical institutes to learn from each other.

One of the newly described triage scales is the Korean Triage and Acuity Scale (KTAS) in South Korea (2). It has been implemented since 2015 and is a five-level system that classifies patients using a combination of variables, including vital signs and chief complaints. 

Triage nurses are likely to triage more conservatively when applying ambiguous triage scales. They try to avoid taking risks as much as possible in clinically ambiguous situations, therefore choosing the mid-point of Likert scale to mitigate any unfavorable consequences (middle effect). Their error remains as low as possible when they choose triage level 3. Triage level 3 is a safe category because it fits a significant portion of incoming patients and it differs only one level from triage level 2 or 4 in case of wrong selection. A recent paper on the validity of KTAS showed that regression to middle effect may occur (2). 

Almost six percent of the intensive care unit (ICU) patients were assigned to triage level 4 and 5 (2). Even if the fairly high proportion of the ICU patients (30.69%) being allocated to level 3 can be overlooked, triage nurses categorizing patients who will be admitted to ICU in level 4 or 5 is not tolerable (2). Therefore, it has to be said that 6% of the ICU patients suffer from under-triage in this subgroup, which deserves further analysis. Almost 59% percent of the general ward (GW) patients were assigned to triage level 3. This implies that triage nurses have mostly assigned GW patients to the triage level 3, which is the most appropriate triage level for patients who need admission to GWs. Choosing level 3 is the most compatible with patients’ acuity in GWs. Almost 58% of discharged (DC) patients from the ED were assigned to the triage level 3, while 35% were placed in level 4 and 5. This shows that a large proportion of DC patients were assigned to level 3, implying that the KTAS is not sensitive enough to assign low-risk patients to level 4 or 5. 

Overall, a high proportion of level 3 patients in all three subgroups shows that triage nurses had a great tendency to assign patients to level 3, implying that regression to the middle effect took place. Regression to the midpoint of a 5-point scale is reported in literature too (3). It is safe for nurses to regress to the middle in case of uncertainty. Kim et al. also showed that 41.6% of patients were assigned to level 3 by the KTAS compared with 38.4% for the emergency severity index (ESI) (4). However, KTAS is more effective than 3-level triage scale, more revisions are needed and further studies are recommended to improve its reliability and validity. In addition, patient influx in level 3 results in other parts of the ED remaining unused. This issue could not be tolerated in the ED because all ED resources must be consumed conscientiously as much as possible to reduce overcrowding. 
